# Novel Thermoreversible Reverse-Phase-Shift Foam With Deployment System for Treatment of Penetrating Globe Trauma in a Newly Described Porcine Model

**DOI:** 10.1093/milmed/usae088

**Published:** 2024-08-19

**Authors:** Ross I Donaldson, Eva Chou, David A Tanen, Jonathan K Armstrong, Oliver J Buchanan, Todd L Graham, Nely N Cristerna, John S Cambridge, Diane Goldenberg, Juliana Tolles, James D Ross

**Affiliations:** Critical Innovations LLC, Los Angeles, CA 90260, USA; Department of Emergency Medicine, David Geffen School of Medicine at UCLA, Los Angeles, CA 90095, USA; Department of Emergency Medicine, Harbor-UCLA Medical Center, Torrance, CA 90509, USA; Department of Epidemiology, UCLA—Fielding School of Public Health, Los Angeles, CA 90095, USA; Ophthalmology Service, Department of Surgery, Walter Reed National Military Medical Center, Bethesda, MD 20814, USA; Department of Surgery, Uniformed Services University of the Health Sciences, Bethesda, Maryland 20814, USA; Department of Emergency Medicine, David Geffen School of Medicine at UCLA, Los Angeles, CA 90095, USA; Department of Emergency Medicine, Harbor-UCLA Medical Center, Torrance, CA 90509, USA; Critical Innovations LLC, Los Angeles, CA 90260, USA; Critical Innovations LLC, Los Angeles, CA 90260, USA; Benchmark Biotech LLC, Portland, OR 97206, USA; Critical Innovations LLC, Los Angeles, CA 90260, USA; Critical Innovations LLC, Los Angeles, CA 90260, USA; Critical Innovations LLC, Los Angeles, CA 90260, USA; Department of Emergency Medicine, David Geffen School of Medicine at UCLA, Los Angeles, CA 90095, USA; Department of Emergency Medicine, Harbor-UCLA Medical Center, Torrance, CA 90509, USA; Benchmark Biotech LLC, Portland, OR 97206, USA

## Abstract

**Introduction:**

The initial management of penetrating ocular injuries is a major sight-threatening problem for both civilian and military medicine. A novel device (Eye-Aid) temporarily tamponades leakage from such injuries while being easy to remove upon arrival to specialized ophthalmologic care. Eye-Aid consists of a protective eye shield with an adhesive backing that connects to a portable canister containing rapidly deployable thermoresponsive foam. The aim of this study was to compare the use of the novel Eye-Aid device to control in a new live swine ocular injury model.

**Materials and Methods:**

Bilateral penetrating ocular injuries were created on 14 male Yorkshire swine in a standardized manner using a 16-gauge needle device to puncture the central cornea and cause a full-thickness wound. Researchers randomized eye intervention side, with the contralateral eye used as paired control. Two minutes after the injury, the eye shield components of the Eye-Aid system, which has a sticky pad for attachment to the skin and a luer-lock for foam deployment, were placed bilaterally. Eight minutes after the injury, foam was deployed for the intervention eye according to the device instructions for use. For the control eye, no additional procedures were performed. Six hours post-injury, end A-scan and intraocular pressure (IOP) were measured. Primary study outcome was change in axial length of the globe. Secondary outcomes were as follows: (1) Presence of full anterior chamber collapse, defined as a lack of measurable anterior lens capsule-reflex (ALC-reflex) on A-scan and (2) change in IOP. Outcomes were analyzed as paired intra-animal data, with intervention and control data for each animal. A paired *t*-test was used to analyze the difference in axial length change and IOP change between treatment groups, whereas a conditional logistic regression was used to analyze dichotomous ALC-reflex outcome and estimate the odds ratio associated with the Eye-Aid device.

**Results:**

A significant difference (*P* < .0001) in mean change in axial length between intervention (−210 μm) and control (−1,202 μm) groups was found. There was a significant difference in ALC-reflex presence, with 79% of eyes having an ALC-reflex in the intervention group, compared to 14% in the control (*P* = .008). IOP remained higher in the intervention group, with a mean change of −1.5 mmHg for the intervention group compared to −4.0 mmHg in the control (*P* = .0001).

**Conclusions:**

This study describes the first development of an in vivo large animal ocular injury model that realistically approximates the emergent time course and pathophysiology of patients with full-thickness corneal open globe injuries. It also gives the first description of using thermoreversible hydrogel foam for such injuries. Eye-Aid was found to be significantly better than control for treatment of such injuries, based on measurements of both structure and pressure. Assuming that the absence of an ALC-reflex demonstrates complete anterior chamber collapse, the Eye-Aid group demonstrated a 79% eye “save” rate compared to only 14% in the control group, as described earlier. This results in a Number Needed to Treat of 3 for this finding. Eye-Aid additionally demonstrated several characteristics that would be beneficial in a device targeted for emergent deployment by non-ophthalmologists.

## INTRODUCTION

The initial management of penetrating ocular injuries is a major sight-threatening problem, with at least 250,000 open globe injuries occurring domestically per year.^1–3^ In the U.S. Military, more than 61,000 incidences of ocular injuries in service members were identified from 2016 to 2019.^4^ In both military and civilian settings, the initial health care provider (e.g., Emegency Medical Technician, paramedic, and combat medic) often does not have the appropriate training and tools to recognize and treat ophthalmologic trauma. Aside from the simple recognition of foreign bodies and chemicals in the eye, the national standard curriculum for Emergency Medical Technician-Basics does not include requirements for orbital trauma diagnosis.^5^ Although the average emergency medical services (EMS) response time in the USA is 7 minutes from receipt of 911 call to arrival on scene,^6^ it can take several hours for patients to receive next-level care from an ophthalmologist. Additionally, despite 10% to 15% of all combat-related traumatic injuries involving the eye, the U.S. Military also has similar limitations that may be compounded by increased delays to definitive ophthalmologic care.^7^

For the past 50 years, the mainstay of first-responder treatment for ocular trauma has been comfort measures and the placement of a rigid eye shield (i.e., Fox Eye Shield) before transfer to the next level of care.^7,8^ In military medicine, current Tactical Combat Casualty Care guidelines simply consist of covering the eye with a rigid eye shield, administration of oral antibiotics, and transfer to the next level of care.^9^ The goal of this protective cover is to prevent additional eye injury from the application of external pressure on the globe that can increase the loss of intraocular contents. Even with proper eye shielding, once collapse of the anterior chamber has occurred, the prognosis for preservation of sight becomes poor, even with subsequent specialty care. Thus, a treatment that can be applied emergently to temporarily tamponade leakage from an open globe injury, which is also easy to remove upon arrival to specialized ophthalmologic care in a ready operating room, would be of great benefit to numerous patients.

To address this important clinical problem, multiple potential treatments have been explored for emergency use by non-ophthalmologists. Traditional treatments with sutures, conjunctival flaps, amniotic membranes, fish scale-derived BioCornea, and related procedures all require use of microsurgical instrumentation and are thus not appropriate for point of injury or en route care.^10–12^ A wide range of bioadhesives have also been explored for the treatment of ocular lacerations. Although tolerated well by the body, the adhesive strength of these products is either too low to tamponade active leakage or too strong, rendering them difficult to remove after application and potentially harmful when used outside a surgical suite.^12^ When considered for use by out-of-hospital providers, these products require eye manipulation and the ability to identify the exact location of any lesion, which are not within existing EMS skillsets. Thus, there is currently no solution available that adequately addresses this important clinical problem.^1^

**FIGURE 1.**
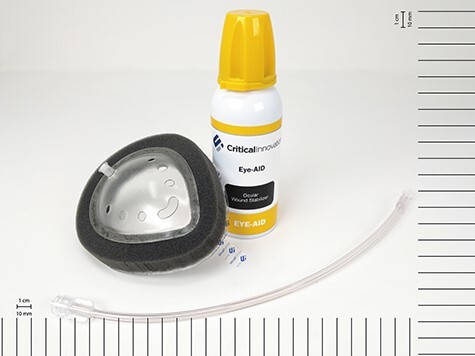
Eye-Aid device before assembly and deployment. Eye-Aid consists of a protective eye shield that connects to a portable canister containing a rapidly deployable thermoresponsive foam.

A medical technology company (Critical Innovations LLC) is developing a new type of investigational device for the emergency management of penetrating eye trauma (Eye-Aid). Eye-Aid consists of a protective eye shield that connects to a portable canister containing a rapidly deployable thermoresponsive foam (Fig. 1). Application of the device requires neither eye manipulation nor diagnostic skills above those typical for current first responders. Once deployed on the eye and warmed to skin temperature, the reverse-phase-shifting foam is designed to self-set into a thick viscous hydrogel barrier. The hypothetical mode of action is that this layer can provide a protective environment that seals injured ocular tissues (e.g., tamponades leaking aqueous humor from a penetrating corneal injury) to provide a temporizing treatment for patients until reaching a higher level of care.

Once reaching ophthalmologic care in an operating suite, the dispensed hydrogel can then be removed from the area via gentle irrigation with cool fluid. Its thermoresponsive reverse-phase-shifting properties mean that the deployed product transitions from a solid gel at skin temperature back into a liquid when chilled (i.e., the opposite of most substances). After swift removal of the hydrogel, the ophthalmologist can then definitively treat the injury.

Here, the investigators describe a pilot efficacy study of Eye-Aid in the management of ocular trauma in swine. We hypothesized that the application of Eye-Aid to a penetrating injury of the anterior chamber of the swine eye would result in decreased loss of ocular fluid in comparison to an eye shield alone as evidenced by measurements of anterior chamber geometry and pressure.

## METHODS

### Regulatory Information

Animal use approval was obtained from the Legacy Research Institute’s Institutional Animal Care and Use Committee and the U.S. Army’s Animal Care and Use Review Office (132-2022 and MT20014.001.e001, respectively). Experiments were conducted in a facility accredited by the Association for Assessment and Accreditation of Laboratory Animal Care at Legacy Research Institute, Portland, OR. Animals were used in accordance with The Guide for the Care and Use of Laboratory Animals.

### Animal Sedation and Preparation

Fourteen male Yorkshire swine (33-45 kg) were obtained from a single source vendor (Premier BioSource). A single sex was selected to minimize intra-group variability and the total animals necessary for the experiment, consistent with the “reduction” principle for animal research. Swine were acclimated a minimum of 3 days following receipt, including the day of receipt. Feed-certified LabDiet 5K99 chow was given twice per day, with rations based on age, weight, and body condition. Water bottles (Lixit) were connected to an automatic watering system and tested daily for proper function. The vivarium’s temperature was set to 21 °C with a fluctuation range of ±1.12 °C, and the humidity level was 30% to 70%. Housing rooms were on a 12-hour light and dark cycle. Each room had more than 10 air changes per hour.

The animals were pre-sedated with 1.1 mg/kg acepromazine and 0.2 mg/kg meloxicam orally and then sedated with 4-8 mg/kg Telazol^®^ (Zoetis) and 0.04 mg/kg of atropine via intramuscular injection. The animals were intubated, mechanically ventilated after induction with isoflurane, and maintained isoflurane at 1% to 3% to maintain a surgical plane of anesthesia. Oxygen was administered at 100%. In addition, animals received 0.005 to 0.01 mg/kg of buprenorphine for analgesia. Maintenance fluids, which consisted of Dextrose in Lactated Ringer’s (B Braun Medical Inc.) 3 mL/kg/h, were administered through an external jugular sheath. Animals were placed in a prone position, and eyes were prepared bilaterally, with swine lid speculums placed to allow for optimal eye visualization. Baseline A-scan (A-1500, Sonomed Escalon) and intraocular pressure (IOP) measurements (Tono-Pen XL, Medtronic) were performed bilaterally.

### Penetrating Injury Creation

A 16-gauge × 1″ needle was prepared by removing its cap, precisely cutting the cap such that it would leave 5 mm of needle exposed and then placing the cap back onto the needle (Supplementary Fig. S1). This was meant to standardize the puncture wound and prevent deeper injury. The eyes were then injured using the exposed needle and puncturing the central cornea to cause a full-thickness wound. The left eye was injured first, and the right injured 60 seconds afterwards. After initial injury, the custom eye shield component of the Eye-Aid system was placed bilaterally. The left eye shield was placed first, 2 minutes after creation of the left eye injury, and the right eye shield was placed second, 2 minutes after creation of the right eye injury. The period of 2 minutes was selected to allow adequate time for eye shield placement before foam deployment.

### Intervention

**FIGURE 2.**
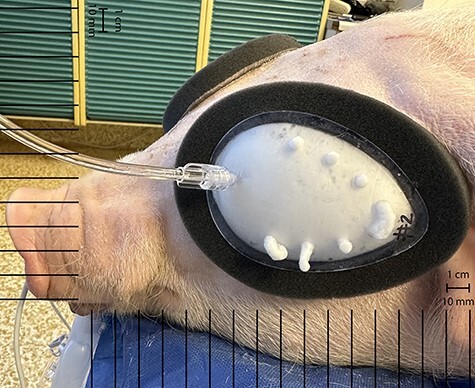
Eye-Aid initial deployment. Researchers randomized eye intervention side, and the animal’s contralateral eye was used as paired control. To simulate time to initial provider, 8 minutes after injury, foam was deployed for the intervention eye according to its instructions for use.

Researchers randomized eye intervention side using the coin flip method, and the animal’s contralateral eye was used as paired control. To simulate time to initial provider, 8 minutes after injury, foam was deployed for the intervention eye (Fig. 2) according to its instructions for use (IFU) (see Supplementary Material). For the control eye, no additional procedures were performed.

### Eye-Aid System Components and Application

The Eye-Aid eye shield has an adhesive pad for attachment to the skin (Fig. 1). The eye shield can then be connected to a portable canister containing the thermoreversible foam via included tubing. To administer hydrogel from the canister into the eye shield, the user actuates the canister’s valve by pressing the stem, similar to deploying foam from a can of shaving cream. The Eye-Aid foam gently flows through the tube and into the eye shield, which normally takes less than a minute. Once the eye shield is filled with foam, dispensing is stopped by removing pressure from the stem. The tube may then be disconnected from the eye shield to facilitate patient movement and transport. While on the body, the dispensed foam settles into a firmer hydrogel over roughly 5 to 15 minutes.

### Animal Monitoring and Study Completion

The animals remained intubated and under a surgical plane of anesthesia throughout the duration of the experiment, with telemetry monitoring including ambient, face (topical), and transesophageal (core) temperatures. Temperatures were recorded for each animal at baseline, 60 minutes, and end of experiment time points. Six hours post-injury for each eye, respectively, the eye shield and foam were removed according to the IFU, and end A-scan and IOP were taken. The animals were then humanely euthanized with 1 mL/4.5 kg of Euthanasia Solution (VetOne).

### Tissue Analysis

Bilateral globes were harvested and fixed in 10% neutral buffered formalin. Subsequently, they were embedded in paraffin, sectioned, and stained with hematoxylin-eosin. A pathologist blinded to treatment groups histologically assessed the samples for evidence of injury. Eyes were examined for evidence of corneal perforation and sagittally sectioned to include the perforation into the histologic section. The presence or absence of evidence of corneal puncture and lens puncture was graded, where 0 = not observed and 1 = observed. Other microscopic changes were graded, as to severity, utilizing a standard grading system, whereby 0 = no significant change, 1= minimal, 2 = mild, 3 = moderate, and 4 = marked. International Harmonization of Nomenclature and Diagnostic Criteria standards are used as the basis of evaluation. Use of numerical grades allows a mechanism to calculate a total lesion score, which can be used to assess prevalence and severity of tissue changes within and between groups. A summary of histopathological findings is shown in Supplementary Table SIII.

### Outcomes and Analysis

**FIGURE 3.**
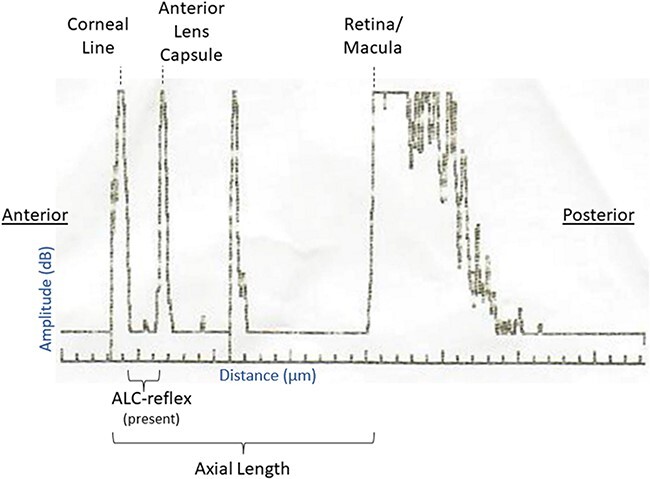
A-scan lines and anatomic correlates. The primary study outcome was a change in axial length of the globe as measured from the A-scan.

The primary study outcome was a change in axial length of the globe as measured from the A-scan (Fig. 3). The secondary outcomes were as follows: (1) Presence of full anterior chamber collapse, defined as a lack of a measurable anterior lens capsule-reflex (ALC-reflex) on A-scan^13^ and (2) change in IOP. Axial length was defined as the distance between the anterior part of the corneal line to the macula, as generated by the A-scan ultrasound.^13^ Presence of an ALC-reflex was defined as a separately distinct and measurable peak between the probe/cornea and the anterior lens capsule with maximum reflective peaks on A-scan.^13^ Anterior chamber depth was defined as the distance between the anterior part of the corneal line to the ALC-reflex, when present.^13^ Outcomes were analyzed as paired intra-animal data, with intervention and control data for each animal. A paired *t*-test was used to analyze the difference in axial length change and IOP change between treatment groups, whereas a conditional logistic regression was used to analyze dichotomous ALC-reflex outcome and estimate the odds ratio associated with the Eye-Aid device.

## RESULTS

All 14 swine were observed to the conclusion of the study with results shown in Supplementary Table SI. Positive intervention was randomized to the right eye 6 times, compared to 8 for the control. Baseline axial length averaged 17,513 µm (SD 519 µm) for the intervention group compared to 17,537 µm (SD 433 µm) for the control. A baseline ALC-reflex was present in all eyes for all animals (100%). Anterior chamber depth averaged 2,656 µm (SD 182 µm) for the intervention group and 2,626 µm (SD 170 µm) for the control. Average IOP was 9.2 mmHg (SD 0.9 mmHg) compared to 8.9 mmHg (SD 1.0 mmHg) for the control. For one animal, IOP was not measured because of device failure. Supplementary Table SII shows an analysis of baseline data. Baseline axial length, presence of ALC-reflex, anterior chamber depth, and IOP were not significantly different between the 2 groups.

Differences in variables at study end are shown in Table I. The primary study outcome was found to be statistically significant, with a difference in the mean change in axial length between intervention (mean change −210 μm) versus control (mean change −1,202 μm) groups (mean of difference 992 μm; 95% CI 796-1,187; Cohen’s *d* = 2.7; *P* < .0001). Additionally, there was a statistically and clinically significant difference in ALC-reflex presence, with 79% (11/14) of eyes having an ALC-reflex in the Eye-Aid intervention group compared to 14% (2/14) of eyes in the control group (odds ratio = 5.5 associated with Eye-Aid, adjusted for within-animal clustering; *P* = .008). Finally, IOP remained higher in the intervention group, with a mean change of −1.5 mmHg for the intervention group compared to a mean change of −4.0 mmHg in the control (mean of difference 2.5; 95% CI 1.5-3.4; Cohen’s *d* = 2.1; *P* = .0001). There was no histopathologic evidence of complications because of foam deployment, with no differences noted between treated and control eyes. Supplementary Table SIV shows ambient, topical, and core temperature data for each animal, recorded at baseline, 60 minutes, and end of experiment time points.

**TABLE I.** Analysis of End of Experiment Results Between Intervention (Eye-Aid) and Control Groups

Final analysis								
Measurement	Eye-Aid^a^	95% CI^c^	Control^a^	95% CI^c^	Mean of difference in pre/post measurements	95% CI for mean of difference in pre/post measurements	*P*-value	Cohen’s *d* or odds ratio
Axial length change (µm)	−210	(−476 to −56)	−1,202	(−1,352 to −1,052)	992	(796 to 1,187)	<.0001	Cohen’s *d* = 2.7
Anterior lens capsule-reflex presence (%)	79%	(49% to 94%)	14%	(2% to 44%)	Not applicable	Not applicable	.0080	OR = 5.5
Intraocular pressure change (mmHg)	−1.5^b^	(−0.8 to −2.3)	−4.0^b^	(−4.7 to −3.3)	2.5	(1.5 to 3.4)	.0001	Cohen’s *d* = 2.1

a Values in these columns are means (*n* = 14, except where noted).

b *n* = 13. For one animal, IOP was not measured because of device failure.

c CI = confidence interval.

## DISCUSSION

Here, to our knowledge, we describe the first development of an in vivo ocular injury model that realistically approximates the emergent time course and pathophysiology of patients with full-thickness corneal open globe injuries, as well as the first description of using thermoreversible hydrogel foam for such wounds. There has been significant research to develop appropriate test models for this important problem with much of it performed on porcine eyes, as swine eye anatomy is thought to be most relevant to penetrating ocular trauma in humans.^2,14,15^ This includes a benchtop model using fresh, whole, porcine eyes,^2,14^ as well as an ex vivo model replicating these injuries using an anterior segment organ culture platform for maintaining ocular tissue.^8,16^

There have been related live animal ocular injury models developed to provide closer approximations to clinical practice,^15^ including an in vivo rabbit model^10^ and a corneal perforation model in Lan-Yu and Göttingen mini-pigs.^11^ However, for all existing models, intervention has been performed immediately after injury. In the USA, average EMS response time is 7 minutes from receipt of 911 call to arrival on scene, with longer response times in rural areas.^6^

As such, our newly described live animal protocol is a notable improvement on previous in vivo models for several reasons. First, it is unrealistic to assume intervention availability immediately after injury, as this only applies to iatrogenically induced surgical intraoperative incisions. Intraoperative lacerations are different from traumatic ocular injuries in many ways.

Second, this new in vivo model is optimally designed to test potential interventions. This new model utilizes larger animals that have similar globe dimensions to adult humans. This is helpful for device testing (e.g., in the current study, the eye shield was placed without modification). Additionally, it allows near one-to-one comparison of volumetric measurements between swine and humans. For example, humans have an average axial length of 22.0 to 24.8 mm,^17^ compared to 17.5 mm for all swine in this experiment. Finally, the use of the A-scan for physical measurement of ocular structures is an improvement on past studies, many of which used IOP as a marker of eye collapse. Although IOP has some utility, it is less reliable and only an indirect marker of the pertinent anatomy, while the A-scan directly measures the clinical finding of import (i.e., collapse of the anterior chamber).

Using this new model, this study provides the first description of using a thermoreversible hydrogel foam for the emergent treatment of full-thickness corneal open globe injuries. Results demonstrate a beneficial treatment effect under all 3 outcome measurements (i.e., axial length, ALC-reflex presence, and IOP change), with statistically significant results in all readings when comparing intervention to control. Assuming the absence of an ALC-reflex demonstrates complete anterior chamber collapse, the Eye-Aid group demonstrated a 79% eye “save” rate compared to only 14% in the control group (OR = 5.5; *P* = .008). This results in an impressive Number Needed to Treat of 3 for this finding.

It appears likely that failure to find an ALC-reflex on A-scan ultrasound equates to complete structural collapse of the anterior chamber, with the corneal and anterior lens capsule-reflex lines merging into one reading. Supporting this supposition, axial length measurements, which include. the depth of the anterior chamber and the rest of the full eye (i.e., cornea to posterior eye), also showed reductions in the axial length. This ranged up to a loss of 1,714 µm in the control group but did not reach the baseline anterior chamber with an average depth of 2,626 µm. This is likely because collapse of the anterior chamber does not happen in isolation. Rather, loss of humor from the anterior chamber can cause deformity of the eye with bulging of the posterior chamber anteriorly, as could macroscopically be viewed on at least some eyes on final exam. Such deformity would keep the axial length loss from fully equating to the starting anterior chamber depth. However, the fact that the axial length had a relatively large and statistically significant reduction is supportive of the overall hypothesis.

Although this model is more clinically relevant than those previously described and benefits from a lack of confounding procedures after intervention, it does not allow for identification of the time course of anterior chamber collapse. Although being a significant improvement over control, a total of 3 eyes (21%) in the intervention group showed no ALC-reflex present (i.e., anterior chamber collapse) at the end of the experiment. Under the current study design, it is not possible to know if this was because the intervention insufficiently sealed these leaks or because it started after those eyes had extravasated too much (i.e., during the 8-minute “time to provider” time). Regardless, we believe that this setup is preferred to taking measurements during this period, which could potentially confound and add bias to the experiment.

Furthermore, the potential for extravasation of aqueous fluid during the 8-minute “time to provider” period also demonstrates why pure “seal” rates should not be the gold standard for identifying lesions amenable to emergency closure. Although we do anticipate future studies with larger and different shaped lacerations, wounds that extravasate fluid at a too fast rate are likely not salvageable even with an intervention that has a 100% seal rate, as they frequently fully collapse before a medical provider could realistically intervene. Thus, this model uses an ideal wound size for potential intervention testing: One that is small enough to realistically allow time to provider yet large enough to result in anterior chamber collapse without intervention.

When considering a potential device for emergent management of ocular injuries, the ability of first responders to realistically deploy it is key to actual improvement in medical practice. Many providers without specific ophthalmologic training are hesitant to manipulate the eye, and devices that require such manipulation are unlikely to succeed in this user group. This is the first study describing utilization of a device that does not require a provider to apply a solution directly to an identified ocular lesion. The use of an eye shield that is similar to the current standard practice, in conjunction with a foaming product, should more easily fit into current skillsets.

Previous research has demonstrated that a critical characteristic for a therapeutic intervention in this area is the ability to remove it upon reaching higher level care, making a thermoresponsive product a potential solution. Based on the temperature data collected during the experiment (Supplementary Table SIV), it is likely that the deployed hydrogel foam was in its solid state for all animals when applied, as the reverse-gel point of the hydrogel foam in this formulation was 20.6 °C (Critical Innovations’ internal benchtop data). At the *T* = 60 time point, average topical face temperature for the animals was 33.9 (±0.64) °C, with a minimum temperature of 32.5 °C. Thus, the deployed hydrogel was likely in its solid state, and we hypothesize, providing a tamponading effect in the Eye-Aid group. Although short-term efficacy was the main focus for this study, it also demonstrated that the Eye-Aid polymer could be easily removed. No differences were noted between control and intervention eyes by a histopathologist masked to treatment group from samples taken at the end of the experiment. This suggests that application and removal of the thermoreversible foam did not cause additional eye injury.

## LIMITATIONS

Although this study showed supportive data for the novel Eye-Aid device in this new model, there are several limitations that limit its findings. First, the study evaluated only 1 injury type. Penetrating ocular trauma can result in a range of corneal pathology, with different patterns and aqueous fluid leak rates. Although the results are promising for the described injury, additional studies would be helpful to confirm efficacy on other injury patterns.

Similarly, this animal model used speculums to maintain animal eyelids in their open positions, while as aforementioned, many of the initial health care providers targeted for this device are hesitant to manipulate the eye (i.e., insert a speculum). Speculum placement was utilized to standardize eyelid positioning and we do not envision utilizing an eye speculum with the device, at least for standard initial use. Although an open eye position is consistent with conscious patients who can voluntarily open their eyes, comatose patients whose eyes are in an open or semi-open state, and patients with significant disruptive eyelid injuries exposing the cornea, it is not applicable to all patients (e.g., conscious patients who are unable or refuse to open their eyelid). For this study, we chose the open position given that it is likely the worst-case scenario for fluid leakage, which theoretically would be higher without the potentially tamponading presence of the eyelid.

Additionally, this study was performed at only one ambient temperature utilizing this thermal-sensitive foam, although extreme ambient temperatures are a realistic possibility in military field environments. Although viable tissue temperature is a much narrower range and the deployed eye shield presumably provides some amount of heat trapping and protection from the external environment, this study did not explore function of the device at different ambient temperatures. Particularly in extreme cold temperatures, the product may become closer to its liquid phase and may not provide as strong of a seal. Future studies would be helpful to further explore the relationship between ambient temperature and device function.

Although this study demonstrated a statistically significant effect for Eye-Aid, no a priori power (sample size) calculation was performed. Future efficacy studies should incorporate an a priori sample size calculation to ensure power for the minimum clinically relevant effect size.

Although a single sex (male) was selected to minimize intra-group variability, future studies will additionally include female animals. This will serve to verify whether results and conclusions apply to females.

Finally, although IOP measurements were significantly reduced in the control versus Eye-Aid intervention group (*P* = .0001), the minimum end IOP in the control group was 4 mmHg (average of 4.9), despite other data demonstrating that the anterior chamber had fully collapsed in the control group in the vast majority of cases (86%). We had expected this value to potentially reach 0 mmHg, reflecting fully hypotony. However, in retrospect, we believe that the Tono-Pen was in these cases measuring the pressure of the bulging posterior chamber through collapsed tissues. This demonstrates why use of the A-scan to determine direct volume measurements is a preferred evaluation methodology in comparison to the use of IOP to indirectly infer anatomic status.

## CONCLUSION

In summary, this study describes the first development of an in vivo ocular injury model that realistically approximates the emergent time course and pathophysiology of patients with full-thickness corneal open globe injuries. It also gives the first description of using a thermoreversible hydrogel foam for such injuries. The animal model has multiple benefits over previously described efforts and may become a standard for testing new therapeutic developments for this important clinical problem. The thermoreversible hydrogel foam deployment device (Eye-Aid) was found to be significantly better than control for the treatment of such injuries, based on measurements of both structure and pressure. It demonstrated several characteristics that would be beneficial in a device targeted for emergent deployment by non-ophthalmologists.

## Supplementary Material

usae088_Supp

## Data Availability

The datasets generated during and/or analyzed during the current study are available from the corresponding author on reasonable request.

## INSTITUTIONAL ANIMAL CARE AND USE COMMITTEE (IACUC)

Animal studies were in compliance with the applicable federal, state, and agency laws, policies, and guidelines for animal protection. Animal experiments took place after receipt of Institutional Animal Care and Use Committee (IACUC) and Animal Care and Use Review Office (ACURO) approvals.

## INSTITUTIONAL CLEARANCE

Institutional clearance was approved.
