# Supplementary material for: Novel Thermoreversible Reverse-Phase-Shift Foam With Deployment System for Treatment of Penetrating Globe Trauma in a Newly Described Porcine Model
Source: Mil Med. 2024 Aug 19;189(Suppl 3):254–61. doi: 10.1093/milmed/usae088 (PMC11332267; doi:10.1093/milmed/usae088)
Supplement: usae088_Supp [file usae088_supp.zip › Table S1.docx]

| **Animal** | **Eye-Aid Randomization** | **Axial Length (µm)** | | | | | | **Anterior Chamber Depth (µm)*** | | | | **Intraocular Pressure (mmHg)** | | | | | |
| --- | --- | --- | --- | --- | --- | --- | --- | --- | --- | --- | --- | --- | --- | --- | --- | --- | --- |
|  |  | **Baseline** | | **End** | | **Change** | | **Baseline** | | **End** | | **Baseline** | | **End** | | **Change** | |
|  |  | **Eye-Aid** | **Control** | **Eye-Aid** | **Control** | **Eye-Aid** | **Control** | **Eye-Aid** | **Control** | **Eye-Aid** | **Control** | **Eye-Aid** | **Control** | **Eye-Aid** | **Control** | **Eye-Aid** | **Control** |
| 1 | Right eye | 17,520 | 17,222 | 17,340 | 15,816 | -180 | -1,406 | 2,774 | 2,534 | 2,510 | - | 11 | 8 | 8 | 5 | -3 | -3 |
| 2 | Left eye | 17,462 | 17,498 | 17,258 | 16,052 | -204 | -1,446 | 2,812 | 2,840 | 2,510 | - | 8 | 8 | 8 | 6 | 0 | -2 |
| 3 | Right eye | 17,694 | 17,544 | 17,924 | 16,322 | 230 | -1,222 | 2,878 | 2,910 | 2,635 | - | 9 | 9 | 6 | 5 | -3 | -4 |
| 4 | Left eye | 17,918 | 17,764 | 17,970 | 16,478 | 52 | -1,286 | 3,020 | 2,840 | 2,864 | - | 9 | 9 | 8 | 4 | -1 | -5 |
| 5 | Right eye | 17,268 | 17,216 | 17,596 | 16,450 | 328 | -766 | 2,826 | 2,714 | 2,818 | 2,510 | 8 | 8 | 8 | 4 | 0 | -4 |
| 6 | Left eye | 18,374 | 17,878 | 18,576 | 16,710 | 202 | -1,168 | 2,616 | 2,474 | 2,492 | - | 9 | 8 | 8 | 5 | -1 | -3 |
| 7 | Right eye | 17,332 | 17,238 | 16,390 | 15,886 | -942 | -1,352 | 2,550 | 2,528 | - | - | 9 | 9 | 7 | 4 | -2 | -5 |
| 8 | Left eye | 16,748 | 17,134 | 17,024 | 16,254 | 276 | -880 | 2,686 | 2,790 | 2,520 | - | 9 | 10 | 9 | 4 | 0 | -6 |
| 9 | Left eye | 18,088 | 18,150 | 17,446 | 17,018 | -642 | -1,132 | 2,580 | 2,566 | 2,636 | - | 9 | 9 | 8 | 6 | -1 | -3 |
| 10 | Right eye | 17,998 | 18,290 | 17,566 | 17,124 | -432 | -1,166 | 2,462 | 2,520 | 2,655 | - | 8 | 8 | 5 | 4 | -3 | -4 |
| 11 | Left eye | 17,228 | 17,244 | 16,084 | 15,530 | -1,144 | -1,714 | 2,534 | 2,422 | - | - |  |  |  |  |  |  |
| 12 | Left eye | 17,146 | 17,282 | 16,780 | 15,910 | -366 | -1,372 | 2,452 | 2,590 | 2,266 | - | 10 | 9 | 9 | 6 | -1 | -3 |
| 13 | Right eye | 16,530 | 16,930 | 16,682 | 16,102 | 152 | -828 | 2,596 | 2,668 | 2,506 | 2,360 | 10 | 11 | 7 | 5 | -3 | -6 |
| 14 | Left eye | 17,880 | 18,126 | 17,604 | 17,032 | -276 | -1,094 | 2,400 | 2,364 | - | - | 10 | 10 | 8 | 6 | -2 | -4 |
| **Average** | | 17,513 | 17,537 | 17,303 | 16,335 | **-210** | **-1,202** | 2,656 | 2,626 | **N/A**** | **N/A** | 9.2 | 8.9 | 7.6 | 4.9 | **-1.5** | **-4.0** |
| **SD** | | 519 | 433 | 667 | 495 | **461** | **259** | 182 | 170 | **N/A** | **N/A** | 0.9 | 1.0 | 1.1 | 0.9 | **1.2** | **1.2** |
| *All cells with no entry ("-") lacked a measurable anterior lens capsule reflex (ALC-reflex) at that time point on A-scan.  **N/A = not applicable  *Results of all 14 swine, observed to the conclusion of the study.* | | | | | | | | | |  |  |  |  |  |  |  |  |

Supplemental Material

Table S1: Animal study data
