# Supplementary material for: Novel Thermoreversible Reverse-Phase-Shift Foam With Deployment System for Treatment of Penetrating Globe Trauma in a Newly Described Porcine Model
Source: Mil Med. 2024 Aug 19;189(Suppl 3):254–61. doi: 10.1093/milmed/usae088 (PMC11332267; doi:10.1093/milmed/usae088)
Supplement: usae088_Supp [file usae088_supp.zip › Table S2.docx]

Supplemental Material

| **Baseline Analysis** | | | | | |
| --- | --- | --- | --- | --- | --- |
| **Measurement** | **Eye-AID™*** | **Control*** | **Mean Difference** | **95% CI*** for Difference** | **p-value** |
| Axial Length (µm) | 17,513 | 17,537 | -24 | (-172 to 125) | 0.7400 |
| Anterior Lens Capsule-Reflex Presence (%) | 100% | 100% | 0% | Not Applicable | 1.0000 |
| Anterior Chamber Depth (µm) | 2,656 | 2,626 | 30 | (-34 to 95) | 0.3300 |
| Intraocular Pressure (mmHg) | 9.2** | 8.9** | 0.3 | (-0.4 to 0.8) | 0.4300 |

Table S2: Analysis of baseline characteristics between intervention (Eye-AID™) and control groups

*Values in these columns are means (n=14, except where noted).

**n=13. For one animal, IOP was not measured due to device failure.

***CI = Confidence interval
