# Supplementary material for: Novel Thermoreversible Reverse-Phase-Shift Foam With Deployment System for Treatment of Penetrating Globe Trauma in a Newly Described Porcine Model
Source: Mil Med. 2024 Aug 19;189(Suppl 3):254–61. doi: 10.1093/milmed/usae088 (PMC11332267; doi:10.1093/milmed/usae088)
Supplement: usae088_Supp [file usae088_supp.zip › Table S3.docx]

Supplemental Material

Table S3: Summary of Histopathological Findings

| **Organ Number -Tissue** | **Group 1 - Treated** | | | **Group 2 - Control** | | |
| --- | --- | --- | --- | --- | --- | --- |
|  | **# Abnormal** | **Mean Group Score** | **Mean Lesion Score** | **# Abnormal** | **Mean Group Score** | **Mean Lesion Score** |
|  | **n = 14** | | | **n = 14** | | |
| **1 – Eye** |  |  |  |  |  |  |
| Corneal puncture  Lens puncture  Congestion, iris, choroid, sclera  Anterior synechia  Corneal edema | 10 | 0.7 | 1.0 | 10 | 0.7 | 1.0 |
|  | 5 | 0.4 | 1.0 | 6 | 0.4 | 1.0 |
|  | 6 | 0.4 | 1.0 | 8 | 0.6 | 1.1 |
|  | 7 | 0.5 | 1.0 | 10 | 0.7 | 1.0 |
|  | 0 | 0.0 |  | 1 | 0.1 | 1.0 |
| Artifact | 14 | 3.0 | 3.0 | 14 | 3.0 | 3.0 |
| Sum – Scores  No Significant Findings: | 28 | 2.0 | 4.0 | 35 | 2.6 | 5.1 |
|  | 2 |  |  | 0 |  |  |
