# Supplementary material for: Novel Thermoreversible Reverse-Phase-Shift Foam With Deployment System for Treatment of Penetrating Globe Trauma in a Newly Described Porcine Model
Source: Mil Med. 2024 Aug 19;189(Suppl 3):254–61. doi: 10.1093/milmed/usae088 (PMC11332267; doi:10.1093/milmed/usae088)
Supplement: usae088_Supp [file usae088_supp.zip › Table S4.docx]

Supplemental Material

Temperature Data

| **Animal ID** | **Baseline Ambient Temp (C⁰)** | **Baseline Topical Temp (C⁰)** | **Baseline Core Temp (C⁰)** | **T=60 Ambient Temp (C⁰)** | **T=60 Topical Temp (C⁰)** | **T=60 Core Temp (C⁰)** | **EOS Ambient Temp (C⁰)** | **EOS**  **Topical Temp (C⁰)** | **EOS Core Temp (C⁰)** |
| --- | --- | --- | --- | --- | --- | --- | --- | --- | --- |
| 20221115-01 | 20.1 | 34.1 | 36.7 | 20.2 | 34.2 | 37 | 20.4 | 34.1 | 37.4 |
| 20221115-02 | 20.1 | 32.4 | 35.4 | 20.3 | 32.5 | 35.5 | 20.4 | 32.3 | 36 |
| 20221116-01 | 20.5 | 33.9 | 36.4 | 20.5 | 33.7 | 36.5 | 20.6 | 33.5 | 36.8 |
| 20221116-02 | 20.5 | 33.7 | 36.3 | 20.5 | 33.6 | 36.6 | 20.6 | 34.8 | 37.6 |
| 20221130-01 | 20.4 | 34.3 | 36.7 | 20.4 | 34.2 | 36.5 | 20.6 | 35.9 | 38 |
| 20221130-02 | 20.4 | 34.2 | 36.6 | 20.4 | 34.5 | 36.8 | 20.6 | 35.4 | 38.4 |
| 20221130-03 | 20.4 | 33.7 | 36.1 | 20.5 | 33.8 | 36.8 | 20.6 | 35.8 | 38.4 |
| 20221201-01 | 19.9 | 33 | 35.6 | 20.1 | 33.5 | 36.1 | 20.3 | 34.9 | 38.5 |
| 20221201-02 | 19.9 | 34.8 | 37.2 | 20.1 | 35.1 | 36.7 | 20.3 | 35.3 | 38.2 |
| 20221201-03 | 19.9 | 33.9 | 36.3 | 20.2 | 33.8 | 36.6 | 20.3 | 36.2 | 38.9 |
| 20221201-04 | 19.9 | 33.4 | 35.5 | 20.2 | 33.3 | 36.2 | 20.3 | 34.9 | 38 |
| 20221202-01 | 20.3 | 34.1 | 36.7 | 20.4 | 34.2 | 37 | 20.5 | 35.1 | 38.3 |
| 20221202-02 | 20.3 | 33.9 | 36.3 | 20.4 | 33.9 | 35.7 | 20.5 | 35.1 | 38.5 |
| 20221202-03 | 20.2 | 34.9 | 37.4 | 20.4 | 34.8 | 37.4 | 20.5 | 34.8 | 38.1 |
| **Average:** | 20.2 | 33.9 | 36.4 | 20.3 | 33.9 | 36.5 | 20.5 | 34.9 | 37.9 |

Ambient, topical, and core temperatures were recorded for each animal at baseline, 60-minute (T=60), and end of experiment (EOS) time points. (EOS = 360 minutes).
