# Supplementary material for: Novel Thermoreversible Reverse-Phase-Shift Foam With Deployment System for Treatment of Penetrating Globe Trauma in a Newly Described Porcine Model
Source: Mil Med. 2024 Aug 19;189(Suppl 3):254–61. doi: 10.1093/milmed/usae088 (PMC11332267; doi:10.1093/milmed/usae088)
Supplement: usae088_Supp [file usae088_supp.zip › IFU Excerpt S1.docx]

Supplemental Material

Instructions for Use followed during this study procedure:

Set up instructions

Check the packaging for damage before opening. Ensure that the product packaging is intact. Do not use the device if open or damaged, or if any of its packaging is broken.
Remove Eye-Aid™ from the packaging.

- Open the Eye-Shield box and remove cover from tray containing the Eye-Shield and Tube, using the peel-tab provided.
- Apply the Eye-Shield.
  - Remove the Eye-Shield and orient it with the Luer connection medially.
  - Remove the adhesive liner using the peel tab.
  - Stick the Eye-Shield onto the patient, with it centered around the eye socket. Apply gentle pressure to insure proper skin attachment.
- Connect the Eye-Shield to the Canister:
  - Open the box containing the Canister and remove Canister from pouch using the tear-notch provided.
  - Remove the pull-tab on the Cap and then remove the Cap by pulling it straight off the Canister.
  - Connect the Tube to the Male Luer connector on the Canister.
- Connect the other end of the Tube to the Female Luer Connector on the Eye-Shield.
- Deploy the Eye-Aid™ hydrogel.
  - To administer hydrogel from the Canister into the Eye-Shield, actuate the Canister’s valve by pressing the Stem laterally.
  - Observe the hydrogel flowing through the Tube and into the Eye-Shield.
  - Once the Eye-Shield is filled with hydrogel, dispensing may be stopped by removing pressure from the Stem.
  - The Tube may then be disconnected from the Eye-Shield, to facilitate patient movement and transport.

Post-Deployment care

- Ensure the Eye-Shield is properly secured to the patient’s skin. The Eye-Shield should remain on the patient during transport or while waiting for higher-level care or additional resources.
- While on the body, the dispensed foam will settle into a firmer hydrogel over roughly 5-15 minutes (depending on room and patient temperature).

Removal instructions

- After reaching higher-level care or when additional resources are available, Eye-Aid™ foam may be removed via gentle irrigation with room-temperature normal saline or sterile water.
- Advanced providers and applicable resources should be available to immediately intervene on the eye injury; the therapeutic benefits of the product are eliminated once removed.
