# Supplementary figures and images for: Novel Thermoreversible Reverse-Phase-Shift Foam With Deployment System for Treatment of Penetrating Globe Trauma in a Newly Described Porcine Model
Source: Mil Med. 2024 Aug 19;189(Suppl 3):254–61. doi: 10.1093/milmed/usae088 (PMC11332267; doi:10.1093/milmed/usae088)

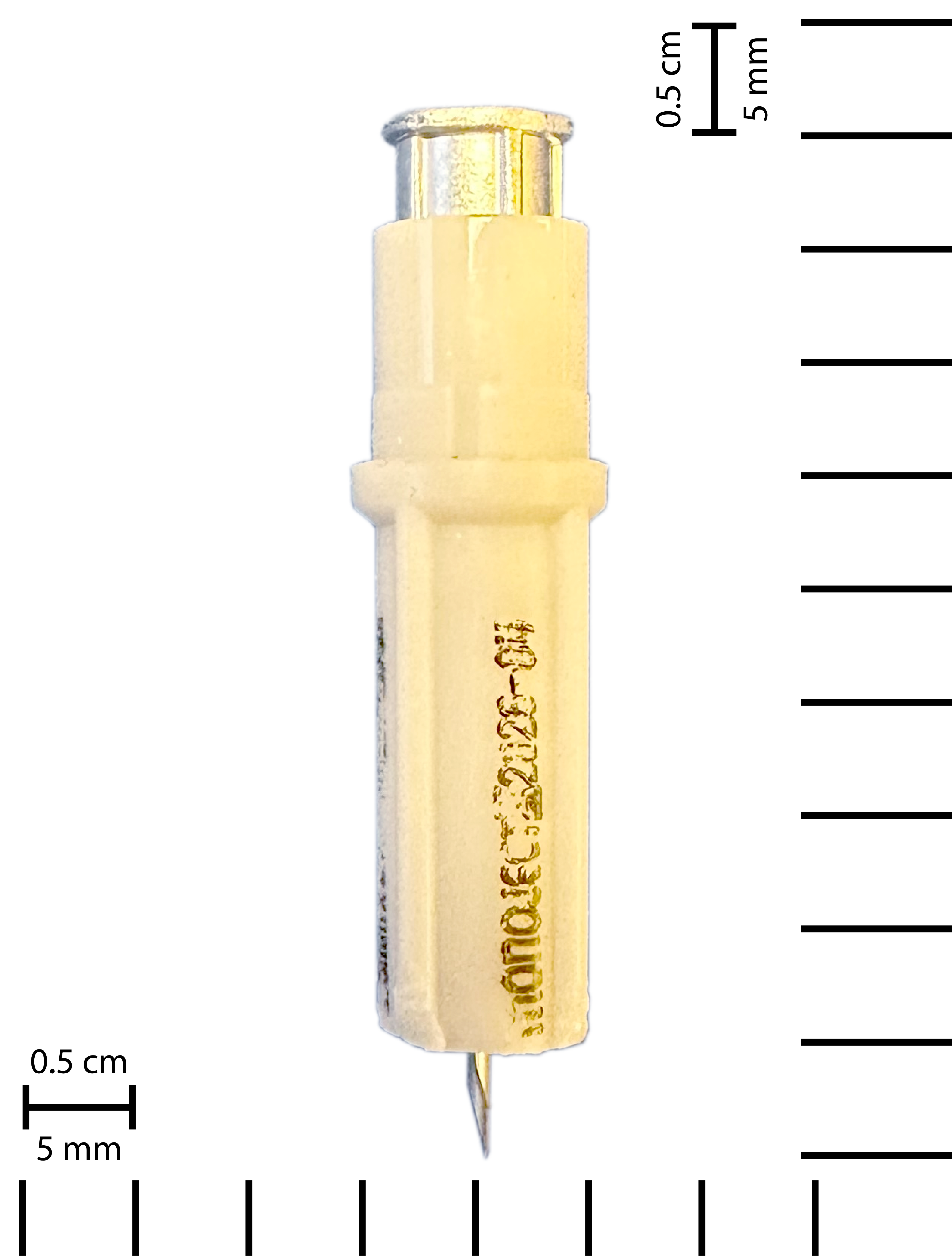

Supplement: usae088_Supp [file usae088_supp.zip › Figure S1.tif]
